# Cultivating the Apprentice-Mentor Model for Minimally Invasive Gynaecology in the Era of Surgically Scarce Training: A Case Report of Laparoscopic Cornuostomy for Interstitial Ectopic Pregnancy by a Trainee

**DOI:** 10.1155/2021/5560309

**Published:** 2021-03-06

**Authors:** Dave R. Listijono, David M. B. Rosen, Sarah Choi, Mujahid Bukhari, Gregory M. Cario, Danny Chou

**Affiliations:** ^1^Sydney Women's Endosurgery Centre (SWEC), Sydney, Australia; ^2^IVF Australia, Sydney, Australia

## Abstract

Over the last few years, there is an apparent growing concern amongst O&G trainees of the inadequacy in exposure to minimally invasive gynaecology surgical training, which has been inadvertently compounded by the more stringent working hour regulations and disproportionately increasing number of trainees relative to surgical volume. Therefore, it is vitally important for trainees to maximise opportunities in the operating theatre and develop autonomy in carrying out more complex surgical procedures. This case report outlines the step-by-step approach of laparoscopic excision of a cornual ectopic pregnancy performed by a trainee under the supervision of a surgical mentor. This manuscript highlights key characteristic traits of a trainee that serve to foster surgical trust and simple but effective steps to foster surgical preparedness.

## 1. Introduction

With the rapidly increasing number of trainees in the specialty of Obstetrics and Gynaecology (O&G) and ongoing restrictions in working hours which, while beneficial from a lifestyle perspective, ultimately limits the volume of surgical procedures and overall experience, the issue of suboptimal surgical exposure amongst trainees is quickly becoming a widespread problem in our specialty, particularly in developed countries [1]. In attempts to overcome this limitation of surgical exposure, trainees have increasingly utilised “lap box trainers” to practice and more recently adapted virtual reality training simulation programs [2, 3], with good effect [4]. Nonetheless, given inadequate allocated time and lack of available curricula, few trainees have truly benefited from this proxy-style practice [5]. Further, a clear disadvantage of simulation training is its deficiency in fostering teamwork and situational decision-making skills [6]. It would therefore seem apparent that real-time primary operation with appropriate guidance remains the most effective form of training to develop surgical competency and autonomy [7, 8]. We hereby present a case report of laparoscopic cornuostomy performed by an O&G trainee, under direct supervision by a gynaecological surgeon consultant, to exemplify the applicability of the apprentice-mentor tenet of surgical education.

Cornual pregnancy (CP) represents a minority (2-4%) of the overall incidence, while carrying the bulk of the morbidity and mortality risk (up to 7 times greater), of ectopic pregnancies [9]. This is in part associated with the anatomical location of the ectopic gestational sac, often proximal to (or within) the interstitial portion of the fallopian tube as it traverses through the muscular wall of the uterus [10] which, in addition to the rich vascular supply of the uterine and ovarian arteries, allows for the tendency to expand prior to rupture, compared to the ampulla segment of the tube, which is commonplace for ectopic pregnancy [9]. With the increasing availability of state-of-the-art equipment and expertise, laparoscopic excision has of late become the first-line approach to surgical management of CP [11]. Compared to conventional laparotomy, laparoscopy has the advantages of improved visualisation, reduced risk of wound dehiscence and blood loss, shorter hospital stay, and faster recovery [12]. Nonetheless, a clear barrier against the uptake of laparoscopic removal of CP remains the technical proficiency required to carry out the procedure [9]. Often touted as a notoriously risky and technically challenging procedure, we present a step-by-step approach to a simple cornuostomy procedure undertaken by an O&G *apprentice* with guidance from a surgical *mentor*.

## 2. Case

### 2.1. History, Examination, and Investigations

**Figure 1:**
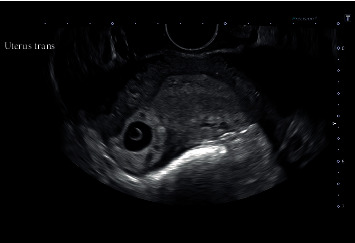
Ultrasound image of a right-sided cornual ectopic pregnancy.

**Figure 2:**
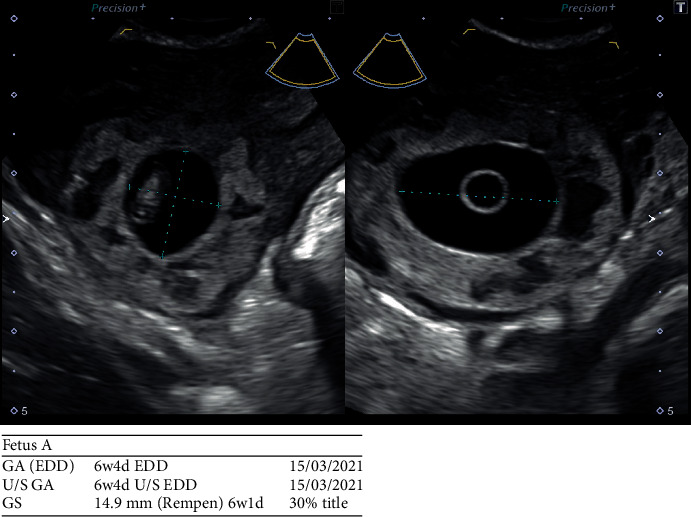
Ultrasound image of the ectopic gestational sac.

A 32-year-old woman presented with an ultrasound-confirmed right cornual ectopic pregnancy (Figures 1 and 2) to the emergency department of a tertiary referral hospital. The patient was haemodynamically stable and opted for surgical removal of the ectopic pregnancy via laparoscopy.

### 2.2. Intraoperative Course

**Figure 3:**
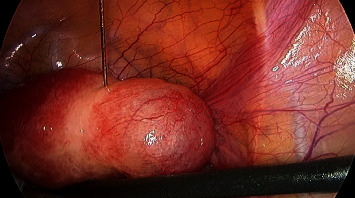
Administration of vasopressin at base of ectopic mass, with good vasoconstrictive effect.

**Figure 4:**
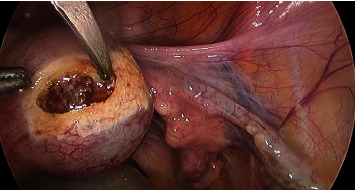
Curvilinear incision to the surface of ectopic mass, exposing underlying trophoblastic tissue.

**Figure 5:**
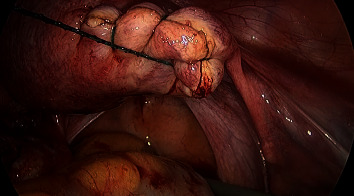
Incision was closed using continuous barbed monofilament suture.

Following general anaesthesia, laparoscopic entry was carried out using the Hasson open technique with a 10 mm umbilical port. Two 5 mm lateral ports and a 10 mm suprapubic port were introduced under direct vision. The right uterine cornua was notably distended by a 4 × 4 cm mass. Following administration of vasopressin (20 IU in 100 mL normal saline) along the base (Figure 3), a single 3 cm curvilinear incision was made on the serosal surface of the mass using monopolar scissors, exposing the underlying gestational sac (Figure 4). The trophoblastic tissue was evacuated, and the specimen was removed via a 10 mm Endo Catch™ bag. Bipolar diathermy was used to obtain haemostasis along the base of the ectopic site. The incision was closed continuously using a V-Loc™ absorbable barbed monofilament suture (Figure 5). The procedure was completed in 45 minutes with an estimated blood loss of 20 mL.

### 2.3. Postoperative Course

The patient was discharged the following day. Histopathology of the specimen confirmed trophoblastic tissue.

## 3. Discussion

Anecdotally, most surgeons would agree that laparoscopic management of cornual ectopic pregnancy is considerably complex and challenging; therefore, it would have been plausible for the consultant to opt as the primary operator in most such cases. The above was performed by a 3^rd^-year O&G trainee for the first time in their career, having previously only seen videos of similar procedures. This case exemplifies the “see one, do one” mantra of traditional surgical education. In the current era, where most O&G trainees are facing unrelenting reduction in surgical volume and exposure, such opportunities are rare and should indeed be cultivated wherever possible. At the heart of surgical training, apprentice-mentor trust is arguably the most important factor in consultants granting trainees increasing levels of autonomy [8, 13]. While *trusting* a trainee to carry out a straightforward low-risk procedure independently may be commonplace amongst supervisors, the extent of supervisor involvement would likely escalate in more complex cases, considering patient safety as the ultimate priority [14]; however, it is in fact in being allowed the opportunity to “safely struggle” that trainees would achieve maximal benefit [13]. To this end, it is imperative for trainees to foster *trustworthiness* and *reliability*.

### 3.1. Construct Surgical Trust through Consistent Display of Ownership and Humility

A recent survey of general surgeons on how they judge a trainee's readiness for operative independence revealed that, aside from the perceived risk of the case itself, the trainee's trustworthiness most likely determines a surgeon's decision to entrust the trainee to do the procedure [8]. However, surgeons rarely referred to trust directly, instead, by observation through a variety of actions and behaviours over time. Survey responses obtained from 120 surgeons identified “integrity,” along with professionalism and work ethic, as the most important attribute they value in trainees [15]. With increasing concern of litigation and fear of criticism, there is a clear shift in surgical trainees away from taking ownership of their decision-making and inevitable mistakes; instead, shifting the focus more towards promotion-associated behaviours [16]. Such etiquette is in fact counterproductive in instilling a sense of integrity [13]. To this end, trainees ought not to fear committing mistakes but rather cultivate the humility to own, and learn from, their mistakes. Anecdotally, there seems to be a tendency, especially within surgical specialties, for humility to be perceived as a form of weakness or lack of self-confidence; however, it would appear that such view is predominantly held by trainees and more junior surgeons [17]. So, make no mistake, humility in medicine implies maturity and emotional resilience—traits that are highly prized amongst surgeons. If only for the development of their character as human beings, trainees should always strive to cultivate these qualities.

### 3.2. I Will Prepare and Someday My Chance Will Come

Be that as it may, a trainee's personality trait would likely only serve in the “initiation of trust” where they are allowed to begin the procedure; ultimately, it is their surgical skills and ability to follow intricate instructions that determine whether the *teacher* adopts the “hands-off” or “takes over completely” approach to supervision [18]. In the operating room (OR), the surgeon educator is constantly faced with the critical balance between supervision, appropriate trainee autonomy, and most importantly patient safety. Studies on factors influencing the level of trainee autonomy in the operating theatre have consistently ranked surgical competency and perception as a “safe proceduralist” as features that would garner sufficient trust for the surgeon to allow the resident to continue with the procedure [8, 19–21]. Therefore, the onus lies on the trainee to place themselves in such favourable positions.

Three fundamental principles that are readily achievable for the trainee include basic understanding of surgical anatomy, familiarity with basic steps of a procedure, and core laparoscopic skills such as knot tying, suturing, and surgical dissection [6]. With working hour restrictions, lack of protected training time, and merciless struggle for operating time amongst trainees, the bulk of these preparations needs to be done outside of working hours and the OR. A simple initial step would be to develop a solid grasp of surgical anatomy through textbooks or focused workshops [22]; indeed, a recent survey of Australian O&G trainees revealed a clear dearth of structured anatomy curricula in the training program [23]. Further, regular review of laparoscopic videos, either open-source or those manually recorded, has also been shown to be useful for skill acquisition amongst gynaecologic residents, particularly in junior learners [24]. However, caution should be exercised in the choice of videos, as there is a clear absence of peer-review process and quality control on most open-source video streaming sites [25]. Trainees should instead be directed towards instructional video tutorials from accredited surgical societies [21]. Finally, to develop psychomotor and visuospatial skills involved in core laparoscopic skills, hands-on *deliberate* practice is indispensable; short of being in the OR and “practicing” on patients, this can be achieved through simulation training [26]. Over the past decade, gynaecological surgical simulation has evolved rapidly, offering a myriad of options, including a virtual reality (VR) laparoscopic trainer, VR robotic trainer, and high-fidelity mannequin simulators [26]. However, the simple and inexpensive laparoscopic box trainer has been shown to be noninferior to more costly options and may be the more worthwhile simulation training of choice for trainees [27].

**Figure 6:**
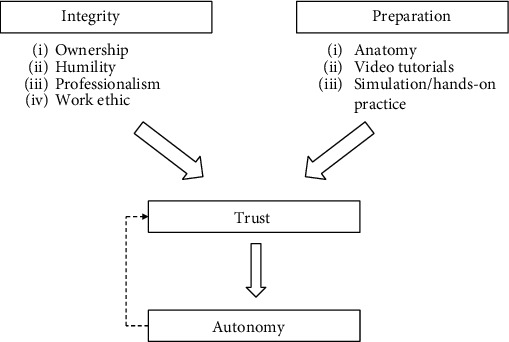
Flow chart of our proposed process towards surgical autonomy for trainees.

Indeed, the trainee who carried out the above laparoscopic cornuostomy was, from a surgical case logbook standpoint, no more than *average* compared to other trainees of their level, but what separates them from the *average trainee* was their personal dedication to minimally invasive gynaecological surgery (MIGS). This drive resulted in them taking initiative in acquiring a basic laparoscopic box trainer as a junior trainee and consistently practiced core laparoscopic skills in their own time, mostly at home. The trainee also undertook surgical anatomy courses from early on in their residency and a Master's degree to further develop relevant theoretical knowledge. To compensate for limited OT time, the trainee obtained laparoscopic videos from MIGS mentors and regularly study the clips and simulate specific movements (e.g., intracorporeal suturing and needle handling) with the box trainer. To maximise OT exposure, they routinely stayed back following their shift *unpaid*, for the opportunity to observe cases by the hospital's dedicated MIGS team. These actions, as summarised in Figure 6, have effectively placed the trainee-in-question in a far more favourable position than their peers, not only from a theoretical knowledge and surgical skills perspective but more importantly, by demonstrating outstanding work ethic and professionalism to prospective MIGS *mentors*—one of whom happen to be the supervising surgeon in the above case.

## 4. Conclusion

The concepts described in this manuscript are by no means groundbreaking, neither is it particularly unique for an O&G trainee to perform laparoscopic cornuostomy. In reporting this case, we endeavour to highlight firstly a relatively *uncomplicated* step-by-step approach to managing a potentially *complicated* surgical case and, in addition, key strategies for current trainees to draw on every opportunity as they navigate through this era of surgically scarce O&G training. The age-old apprenticeship model of gynaecological surgical training is very much alive and well, and it is up to trainees to utilise available tools to optimise training outside of the OR and align themselves with character traits to engender trust in their surgeon mentors.

## Data Availability

Data are available from the corresponding author on request. Please email Dr. Dave Listijono at d.listijono@unsw.edu.au.
